# Implementation of postnatal care for HIV-positive mothers in the Free State: Nurses’ perspectives

**DOI:** 10.4102/phcfm.v11i1.1776

**Published:** 2019-04-25

**Authors:** Lumka Mangoejane, Mokholelana M. Ramukumba

**Affiliations:** 1 Maternal, Child and Women’s Health Unit, Free State Department of Health, Bloemfontein, South Africa; 2 Department of Health Studies, University of South Africa, Pretoria, South Africa

## Abstract

**Background:**

Postnatal care (PNC) provides the opportunity for protecting the lives of women infected with human immune deficiency virus (HIV) and their babies. The prevention of mother-to-child transmission of HIV (PMTCT) guidelines provide a framework for implementation of PNC. There has been no empirical evidence on how the nurses at the clinic level implement these guidelines. In addition, there are reports that PNC has been neglected in South Africa.

**Aim:**

The study aimed to explore the implementation of PNC for HIV-positive women, by explicating nurses’ views regarding their practices.

**Setting:**

The study was conducted in 2015 at three clinics at Mangaung Metro Municipality in the Free State.

**Methods:**

A qualitative, evaluative case study was conducted to provide a detailed account of the implementation of PNC, using 2015 PMTCT guidelines as a framework for evaluation. Eighteen key informants participated in three focus groups. Data were reviewed through direct thematic analysis.

**Results:**

Four themes emerged from data analysis, namely, guidelines as an empowering tool, implementation of HIV guidelines, perceived successes and challenges of postnatal HIV care, and measures to strengthen postnatal HIV care services. The study found that nurses interpreted and used guidelines to direct their practice. However, there were challenges and some successes.

**Conclusion:**

It was concluded that nurses had a good understanding of the guidelines provided for their practices and implemented them with various levels of success. Effective management of HIV-infected women during the postnatal period requires well-designed multidisciplinary collaborations, adequate resources, continuous professional development programmes, a high level of competence and confidence.

**Keywords:**

community health centre; HIV-positive women; nurses; primary healthcare; postnatal HIV care.

## Introduction

Maternal health remains a priority of global public health, and the disparities are growing between the developed and developing countries.^1^ One of the important health indicators for South Africa and other developing countries is maternal mortality. Measures of maternal mortality reflect women’s access to and use of healthcare services.^1^ Primary healthcare (PHC) re-engineering is one of the measures to strengthen PHC through the district health system. Health systems should be strengthened in order to produce better health outcomes and to achieve long and healthy lives for all South Africans.^2^ To implement the PHC re-engineering, the National Department of Health has focused on three priorities or steams: the PHC ward-based outreach teams (WBOTs), strengthening school health services and district-based clinical specialist teams with initial focus on maternal and child health.^3^ Primary healthcare in South Africa faces many challenges such as inconsistencies in the quality of care delivered by health professionals, the burden of disease, health worker shortages, suboptimal supervision and support and underfunding in public health challenge innovations at the PHC level.^4^

The national consolidated guidelines for the prevention of mother-to-child transmission (PMTCT) and the management of human immune deficiency virus (HIV) in children, adolescents and adults provide a framework on how to respond to and to manage HIV-infected individuals.^5^ In an effort to reduce maternal and infant deaths, postnatal care (PNC) for women infected with HIV provides the opportunity for protecting the lives of both mothers and their babies by optimising HIV management. Antiretroviral treatment (ART) is often initiated by a professional nurse, and this is known as nurse-initiated and managed ART (NIMART). Nurses in the postnatal period are confronted with a variety of HIV-related situations. Some women are already on ART, while others’ HIV status is not known, especially the ‘unbooked cases’. There is also an issue of HIV-exposed infants who need to be properly managed to maximise effective management of HIV-positive women during this period. How the nurses provide care to these mothers and their infants is of paramount significance in the reduction of mortality in the postnatal period.

The Free State Province has 232 PHC clinics that render maternal and child health services as a PHC core package. Human immune deficiency virus care and management is integrated into routine care. Professional nurses are trained on NIMART to scale up initiation of women on ART. Uptake of ART among HIV-infected pregnant women is above 80%.^6^ Postnatal care is provided in all facilities, but at 6 weeks utilisation has been found to be low. However, there was an increase in 2014, with 81.3% of women receiving PNC within 6 days after giving birth^6^ as compared to 79.7% in 2011. In spite of all these efforts, non-pregnancy-related infections, including HIV and AIDS, still remain the top causes of maternal deaths in the Free State.^7^ Women with low-risk pregnancy receive antenatal care at the primary health clinics and deliver in the maternity-obstetric-unit. Human immune deficiency virus infection without complications is not classified as a high-risk factor. Postnatal follow-up is provided at a PHC setting nearer to patients’ place of stay. Community health centres provide comprehensive primary care services including deliveries. These facilities provide appropriate and accessible healthcare services to the communities.

The 2015 PMTCT consolidated guidelines stipulate that women who test negative should be retested after every 3 months, as it has been discovered that about 4% of women who initially tested negative later test positive for HIV. Women who test positive for HIV within 1 year after giving birth should be initiated on treatment irrespective of the CD4 count.^4^ An investigation of PNC services to HIV-positive mothers and their HIV-exposed infants in Swaziland^8^ found gaps in the implementation of policies and the quality of PNC. The study found that 11.3% of HIV-positive women were not given information on the importance of co-trimoxazole prophylaxis, which put them at risk of developing opportunistic infections. One study^9^ identified that women were not given enough information during pregnancy and after birth, which affected their confidence in self-care and care of their babies. This implied the need to improve the quality of information given to women during PNC especially before discharge. There is evidence that women are motivated to address health issues during pregnancy and PNC.^10^ Therefore, the postnatal period presents an important intervention opportunity. The study sought to explore implementation of PNC to HIV-infected women in the Free State by explicating nurses’ views regarding their practices. The 2015 PMTCT consolidated guidelines were used as a framework.

There are reports that PNC has been neglected in South Africa.^11^ Performance on PNC within 6 days has varied across provinces, with the Free State having performed higher than other provinces at 79.7%.^12^ Interventions to prevent mother-to-child transmission are critical to reduce infant HIV infections and child mortality.

## Methods

### Study design

A qualitative, evaluative case study design was used to provide a detailed account that involved description of implementation of PMTCT guidelines to postnatal HIV-positive clients. Qualitative research is an enquiry that seeks to explore human experience of the studied phenomenon for understanding of the participants’ actions.^13^ It allowed the researchers to build a holistic picture of the implementation of PNC in a natural setting.^14^ In qualitative methods, the researcher can increase the depth of understanding that he or she may gain from the experience through exploration.^15^ The evaluative case study was the preferred study design as it allowed examination of specific instances such as interpretation and implementation of HIV national guidelines, thus illustrating the complexities of the situation of managing HIV in PNC. The heuristic quality of a case study, such as the ability to offer reasons for a problem, and providing the opportunity to evaluate what worked and what did not; made it most appropriate for this study.^16^ This type of research design is most valuable in exploratory research.^17^

### Study setting

The study was conducted in the three community health centres in Mangaung Metro Municipality, which provide comprehensive PHC services including maternal, child and women’s health. The researchers picked sites that yielded the most information and had the greatest impact on the development of understanding regarding implementation of PNC for HIV-positive mothers.

### Participants and sampling

The population comprises nurses registered with the South African Nursing Council working at the PHC clinics, with various ethnic backgrounds. A non-probability, criteria purposive sampling method was used to select participants, which allowed selection of a homogenous group to enable focused enquiry. Nurses who were enrolled as nursing assistants working in the postnatal units were excluded from the study. Because this was an evaluative case study, the approach integrated elements of typical case, homogenous and criteria sampling.^18^ The inclusion criteria were nurses trained in ART and working with postnatal HIV-infected mothers. The rationale for this approach was to describe and illustrate what is typical in the PNC unit serving HIV-positive mothers, to minimise variation by recruiting a homogenous group and use predetermined criteria to select nurses who have the necessary knowledge and experience of postnatal HIV care. This method allowed an in-depth understanding of the implementation of PNC using PMTCT guidelines as a point of reference.^13^ The PMTCT guidelines were used to frame data collection, as nurses’ practice in HIV management is largely controlled by HIV policy.

### Data collection

Data were collected in October to November 2015 using a semi-structured interview guide. Three focus groups (FGs) with five to six participants in each were conducted at three clinics. The procedures followed included setting rules, keeping discussions confidential and respect for each participant’s viewpoints.^18^ The first author collected data using digital audio recorder. The participants were asked about their interpretation of the PMTCT guidelines, how they managed postnatal HIV-infected women, their achievements since implementing the guidelines, challenges and suggestions to address those challenges. The audio recordings were shared with M.M.R. to determine the need for additional data. The process continued until no new information emerged, and this stage signified data saturation. The researchers explicated their beliefs about the phenomenon; these were written down and kept as a separate log, prior to data collection. The researchers remained open to data emerging from the participants by bracketing their thoughts and perceptions.

### Data management and analysis

The research analysis steps described in Creswell^19^ were followed. These included transcription, immersion in data, coding, developing categories and comparison across categories. An inductive thematic analysis was used to review and identify common issues that recur, and these were summarised in narrative form. Audio recordings were transcribed verbatim and typed using the Microsoft Word program. The transcription of data occurred after each FG interview, followed by a short description of each group’s data and preliminary analysis. The period of immersion included reading of transcripts over and over again. Similar and different views from the different FGs were merged; this was followed by searching across the data sets to find repeated patterns of meaning. Data were then summarised using codes and compared to establish the relationships among the different categories. The researchers examined the interpretations and implementation of PNC to identify themes. Themes were consolidated to develop meanings.

### Trustworthiness of the study

The researchers used audit trails, member checking and bracketing to enhance the confirmability of the research results. Continuous checks were built into the data collection process by using participants’ verbatim accounts and using the audio recorder and member checks to ensure confirmability. Notes obtained from the fieldwork were reviewed and the voice recording was listened to repeatedly, ensuring that the data represented participants’ views and actual practice. The first author sought confirmation from participants that the interpretations were their true reflections.

Credibility was promoted through prolonged interaction, remaining in the field until saturation of data was attained. Reflexivity and bracketing were used to set aside views, existing knowledge and preconceived ideas about care of HIV-positive women during the postnatal period.

The research design, methods and their implementation, data collection process and procedures used by the researcher in the study were described in detail. The researchers selected information-rich participants such as nurses who had been trained in HIV and who managed HIV-positive women in postnatal clinics. Data were collected until data saturation occurred. The thick descriptions of data were generated on the premise that, in similar contexts and conditions, the results could be transferable.

### Ethical considerations

Ethical approval was obtained from the University of South Africa Health Studies Higher Degrees Committee, College of Human Studies (HSSHD/ 401/2015), and the Free State Department of Health. Permission and informed consent from the nurses were obtained prior to the commencement of the study. The purpose of the study was explained prior to data collection. All participants were above 18 years and were eligible to give informed consent. They were made aware that they were not forced to participate in the study and that they had the right to withdraw at any time, not answer questions that they felt violated their privacy and withhold information without being penalised. The participants consented to the use of a digital audio recorder.

The transcripts and recordings were kept in a safe place using passwords to protect the electronic files. Paper files were stored in a locked cupboard to prevent unauthorised access. Codes were used instead of names to ensure anonymity.

## Results

Four major themes emerged from the data. These were: guidelines as an empowering tool, current HIV care practices, successes of postnatal HIV care, challenges and measures to strengthen postnatal HIV care services (see Table 1). The first two themes were related to the views of the participants regarding interpretation of the guidelines and implementation of postnatal HIV care. The third theme was based on the recommendations on how to strengthen services provided to HIV-infected women. The PMTCT national guidelines provided the framework for the interpretation and conclusions.

**TABLE 1:** Themes and subthemes.

Theme	Subtheme
**Guidelines as an empowering tool**	Framework for HIV management Increasing HIV treatment opportunities Holistic approach and more focus on women
**Implementation of HIV guidelines**	Care of HIV+ mothers and HIV-exposed babies Discharge of HIV+ mothers Drug management Collaboration with WBOTs
**Perceived successes and challenges of postnatal HIV care**	Quantifiable outcomes Low utilisation of postnatal care Resources (staff and drugs) Booking system and referrals Lack of uniformity in HIV care
**Measures to strengthen postnatal HIV care services**	Further improvement of care Increasing adherence to treatment Continuous professional development Integration of services

HIV, human immune deficiency virus; WBOTs, ward-based outreach teams.

### Guidelines as an empowering tool

Participants described guidelines as a general framework for the management of HIV-infected women and their babies. They understood the guidelines as a tool that enabled them to initiate treatment to HIV-positive clients at the point of diagnosis. They also believed that it was the government’s approach to show its commitment to reducing the infection rate, increasing access to treatment and providing protocols for health professionals to manage HIV effectively. However, it appeared that the guidelines were not detailed enough as in some instances they used their nursing background to offer specific care. This is supported by the quote that follows:

‘The guidelines are general and do not provide for individual care. During implementation, we adopt them to suit specific scenarios. At times, we have to go out of the parameters of the guidelines and use our nursing knowledge to give care, because I understand guidelines to be broad.’ (FG2, 39 years old, nurse)

Participants explained that in general, the 2015 guidelines are more comprehensive and there is more focus on women in that the services have been expanded to include cervical cancer screening 6 weeks post-delivery and then yearly, screening for tuberculosis (TB), family planning and the management of sexually transmitted infections. The following statement represents their views:

‘In the new provisions, the fourth prong of PMTCT puts an emphasis on holistic management of HIV-positive women, including health education on how to care for their babies and management of STDs.’ (FG1, 30 years old, nurse)

The guidelines were also interpreted as an attempt to minimise missed opportunities by not only focusing on PNC but also tracking the history of the woman from antenatal care and include those who tested HIV negative during pregnancy. Participants showed understanding of the treatment cascade by indicating that HIV-positive women who were not diagnosed during antenatal care or given prophylaxis were at risk of developing opportunistic infections during the postnatal period:

‘The change is about testing for HIV. Previously HIV-negative women were retested at 32 weeks irrespective of when the initial test was done; now it is after every three months to minimise missed opportunities. There is a chance for follow-up from pregnancy.’ (FG3, 40 years old, nurse)

### Implementation of HIV guidelines

Nurses indicated that they followed guidelines, offered comprehensive care to these mothers and encouraged breastfeeding within an hour of delivery. All assessments and screening were carried out on mothers to ensure quality care. Human immune deficiency virus-positive women were discharged within 6 hours if there were no complications. All HIV-exposed babies were tested for HIV and medication given accordingly. The repeat test was performed after 10 weeks. As stated by a participant:

‘Babies born from HIV-positive mothers are given nevirapine syrup at birth according to the dosages in the guidelines. In case the mother is diagnosed during labour we also give the baby AZT [*azidothymidine*], which she will take together with nevirapine. We advise the mother that the baby must drink AZT until the nurse discusses the PCR [*polymerase chain reaction*] results with her.’ (FG3, 30 years old, nurse)

What featured frequently was the various aspects of health education and counselling that nurses provided. The emphasis was on self-care, adherence to treatment for herself and the baby, contraception and child spacing, nutrition, safe infant feeding and monitoring of danger signs. However, they also expressed limitations and inadequacy with counselling services that they offered. It is generally expected that nurses at these facilities will provide counselling on various issues such as HIV and couple counselling. However, they are faced with a high workload of managing HIV-positive mothers and their exposed babies and have to rely on lay counsellors for counselling. Statements by participants:

‘Mothers are informed on the importance of follow-up care at six days and six weeks. At six days, they are counselled on infant feeding; they are done breast examination to check for signs of infection as it may increase the chances of mother-to-child transmission of HIV.’ (FG2, 39 years old, nurse)

‘We have patient overload and proper counselling on various issues such as emotional and nutrition should be handled by appropriate professionals.’ (FG1, 30 years old, nurse)

According to the nurses, the 6-week visit entails growth monitoring of the baby, prescribed medication and feeding. Mothers are monitored for disease progression by having a CD4 count and World Health Organisation clinical staging. Human immune deficiency virus -infected breastfeeding women are initiated on fixed-dose combination or zidovudine immediately irrespective of the CD4 count. Participants also believed that much could be done to increase confidence levels in ART initiation. The majority claimed that WBOTs are a resource used to trace and follow-up postnatal patients in communities. During the household visits they identify patients with complications and are referred to the facility for further management. Nurses indicated that all infants are given immunisations according to the Expanded Programme on Immunisation schedule. However, babies whose mothers have active TB are given isonicotinohydrazide prophylaxis; if the mother has been on TB treatment for less than 2 months, the Bacille Calmette-Guerin (BCG) vaccine is delayed until she has completed TB treatment. The nurses acknowledged that effective PNC requires patients to take responsibility for and ownership of the care and take the initiative, such as early antenatal registrations, voluntary testing and HIV disclosure. Early booking or registration was related to early identification of risks and early initiation of treatment that will be continued during the postnatal period. As stated by participants:

‘At six weeks, HIV-positive mothers are given Bactrim [*co-trimoxazole*] to give to the babies. If the mother has opted for breastfeeding, of which most do, they are taught to give it until after six weeks when the baby has stopped breastfeeding … those who are not breastfeeding are advised to stop when the baby tests PCR negative. Babies whose PCR test result is positive continue with Bactrim to prevent opportunistic infections.’ (FG2, 40 years old, nurse)

‘Different stakeholders have a significant role during PNC; WBOTs create a link between us and the communities. However, women must realise that their participation determines the success of HIV management.’ (FG3, 36, years old, nurse)

### Successes and challenges of the postnatal HIV care

Nurses indicated that their practices yielded some quantifiable positive outcomes, such as an increased number of patients initiated on ART, a decrease in the maternal deaths and the rates of PCR positive results. Participants acknowledged that, although there were measurable successes, there were still challenges that needed to be addressed:

‘The changes in the eligibility criteria enable us to initiate more women on Antiretrovirals [ARVs]; in our district, you hear of few AIDS-related deaths. HIV transmission to the babies has decreased. We rarely receive positive PCR results. If we do, it will be one after a long time.’ (FG1, 30 years old, nurse)

Some of the challenges highlighted included low utilisation of PNC, inadequate resources, lack of clarity of referral procedures and shortage of skilled staff as not all nurses working in the postnatal unit are trained in PMTCT:

‘Some patients come late for postnatal care and others do not come at all. As a result, our postnatal uptake within six days is affected and care is delayed. A small number of women, who did not book for antenatal care, come for deliveries, often do not come back for postnatal care and therefore do not receive postnatal care services.’ (FG2, 39 years old, nurse)

‘Some of the drugs have been out of stock for some time. If patients fail on the first-line regimen, it is difficult to switch to the second-line regimen.’ (FG3, 40 years old, nurse)

‘The gaps in our referral system make it challenging to provide continuity of care to the patients. When we refer patients to the hospital for delivery, there is no back referral; we are not sure whether they go back to their primary healthcare clinic nearest to their place of stay or skip treatment.’ (FG2, 39 years old, nurse)

### Measures to strengthen postnatal HIV care services

Participants acknowledged that more still needed to be done to improve the healthcare services to HIV-positive women in the postnatal period. They re-emphasised the importance of a holistic approach that also includes psychological, socio-cultural and economic aspects, as these also determine adherence and the progression of the disease. They felt that policy needed to strengthen provider-initiated counselling and testing during pregnancy. Testing should be extended to WBOTs, for early identification of HIV-positive women. They claimed that the earlier the client is tested and initiated on treatment during pregnancy, the better the outcomes for PNC:

‘HIV-positive women should receive proper testing, treatment and counselling from the antenatal period; we should also improve collaboration with social welfare services. There should be focus on addressing their fears and reassure the women on the possibility of living positively. WBOTs’ role could be expanded to include testing at home.’ (FG1, 30 years old, nurse)

Participants recommended that nutrition form part of the HIV management strategies. They felt that facilities must have full-time dieticians to counsel mothers on nutrition as nurses often do not have time. Currently, there are only visiting dieticians. They also recommended that women who test HIV-positive should be routinely offered nutritional supplements:

‘Nutritional support, drug readiness training, and proper follow-up from antenatal period should be reinforced in the HIV management strategies to enhance adherence.’ (FG3, 42 years old, nurse)

Nurses recommended that women attend drug readiness training that will prepare them psychologically and be ready for treatment. They also believe that, while the guidelines stipulate that women be initiated at the point of HIV diagnosis, they should be followed up and supported throughout the PMTCT cascade to improve adherence:

‘Clinics need to establish support groups to increase adherence and encourage clients to have treatment buddies for support.’ (FG2, 39 years old, nurse)

Participants indicated that there are differences in public and private sectors in HIV management, and that creates challenges in the continuation of care to clients who present in public healthcare facilities. They indicated that the policy should be applicable to both private and public sectors:

‘Doctors in the private sector put patients on different regimen from that used in the public sector; some of these patients end up in the public service and it is a challenge to manage them.’ (FG1, 49 years old, nurse)

Nurses recommended that there be continuous training on NIMART and that mentorship be provided to improve confidence in initiation of ARVs and knowledge of HIV management during the postnatal period. They further indicated that they need continuous updates on the latest developments to ensure that they provide accurate information:

‘There should be mentors in all facilities to support nurses on initiation on ART. In this way, people will be confident to initiate treatment. We need to be kept up to date so that we give women the correct information.’ (FG3, 40 years old, nurse)

## Discussion

The study was based on the premise that in order to fully understand the policy outcomes, the perspectives of the key participants in public policy implementation should be explored. Consequently, for any care to be effectively implemented, the professionals at the forefront, in this case nurses, must have a good understanding of the relevant policy and its provisions. It is common assumption or knowledge that developing countries often face challenges with implementation of public policies. In this study, nurses subjectively shared their views and understanding of what the PMTCT guidelines meant to them as professionals and their work in the PNC unit.

The results showed similar, but slightly varied explanations. Some nurses understood them as a broad framework for HIV management, which is aimed at increasing treatment opportunities, while a few understood them to be general directives or protocols to initiate ART. This implies that implementation of national HIV guidelines will be largely influenced by nurses’ knowledge and self-confidence in HIV management. All nurses believed that the guidelines were comprehensive in that screening procedures were included, such as Pap smear and TB. There were no divergent interpretations among the three groups. They were also aware of the provisions for treatment for women who tested positive at different stages, for example, during labour and postnatal periods.

The nurses mainly followed and applied the guidelines in their current practices. They provided comprehensive HIV care to both mothers and HIV-exposed children. They understood the significance of PCR at birth and appeared to manage the neonates adequately. Limited health education, advice on nutrition and counselling were provided as they did not feel they were adequately prepared to give full counselling, including advice on nutrition, as there were no professional counsellors employed in these facilities. The increased workload prevented them from offering adequate counselling services; they relied on lay counsellors. Counselling is important in managing HIV to improve retention in the programme. Their nursing background supported areas where they experienced knowledge gaps. The discharge planning and care was also viewed as important, as it is a critical period for the mother and baby, because most maternal deaths occur during this period.^8^ However, it has to be acknowledged that in a limited-resource context, management of HIV creates a higher workload on already stretched professionals.^8^

Though the facilities had access to community-based resources, the study found that there was still a need to strengthen collaborations to improve retention in care. Human immune deficiency virus-exposed babies were given immunisations as per schedule. However, this study could not establish the follow-up and management of exposed infants at 6 weeks because that is done at the immunisation clinic in the same facility. The study identified the finding as a challenge to proper follow-up. Tuberculosis and cervical cancer screening are integrated in the postnatal HIV management; they treat women who screen positive for TB, and those who screen negative are put on TB prophylaxis. The practice is in line with the guidelines.^5^ This is supported by Ndwinga et al.,^20^ who posit that PNC offers a platform for TB screening and increased treatment of TB-infected patients. Ndwinga et al.^20^ also found that the integration of TB screening enhanced the quality of PNC.

Clinical guidelines promote interventions, reduce morbidity and mortality, improve consistency of care and improve quality of life. Clinical guidelines put emphasis on provider-initiated counselling and testing routinely to encourage patients to know their status.^4^ Participants acknowledged the challenges involved in early detection, in that some patients volunteer or agree to be tested but others who test HIV-positive do not bring their partners for testing. The collaboration with the WBOT was intended to encourage women to agree to testing and improve adherence. Nurses followed the PMTC guidelines and demonstrated full understanding of what is required. They understood when and in which instances they were to give babies BCG, co-trimoxazole and nevirapine. In addition, the stage of ART initiation in relation to CD4 count was well within the guidelines. They showed confidence in the implementation of the national HIV guidelines. This finding is supported by Geogeu et al.,^21^ who indicated that the nurses’ understanding of the guidelines and their use has a positive influence on their clinical confidence. Pillay and Barron^2^ supported the findings and confirmed that HIV-exposed infants are put on nevirapine and zidovudine, depending on the duration for which the mother has been on ART. Co-trimoxazole is given to prevent occurrence of opportunistic infections, and the duration of treatment is guided by PCR results and whether or not the mother is breastfeeding. Eley^22^ argues that in South Africa, identifying HIV-exposed infants remains a challenge. However, the birth DNA PCR testing and early ART initiation could minimise mortality between 1 and 3 months. Similarly, Sprague et al.^23^ confirmed that in Gauteng, they found delayed HIV infant testing as one of the weaknesses in the implementation of the PMTCT. In this study, nurses carried out a PCR test on all HIV-exposed babies, implying that with good interdisciplinary support and availability of drugs nurses can play a significant role in the prevention or reduction of infant mortality.

Nurses described some measurable outcomes of their practices. They indicated that there was a decrease in babies testing positive for PCR and an increase in the number of women they initiated on ART – their goal was to achieve 100% coverage. This shows commitment on their part to render effective service. Over the past 3 years, there has been constant improvement in ART initiation across the Free State; however, the province has not been able to attain the 95% target. The facilities in the study have maintained ART initiation in pregnant women above 90%, with the exception of Facility B, which dropped significantly in 2014. Reasons associated with the underperformance were non-availability of drugs and lack of skills to initiate ART in pregnancy. Dlamini^8^ also found that in facilities where there is no pharmacist or pharmacy assistant, drug management is a challenge. Failure to initiate ART during pregnancy defeats the goal of reducing the viral load to undetectable levels and improving the CD4 count. The success of ART for improving maternal well-being and reducing mother-to-child transmission of HIV depends on the adherence to treatment throughout the continuum of care.^21^ The low utilisation of postnatal care, inadequate resources and referral systems were cited as critical issues. These are countrywide challenges that confront the PMTCT service and the healthcare system as a whole.^24^

Participants believed that provision of PNC to HIV-positive women was a complex process and its success depended on adequate management of the challenges cited. Several recommendations were proposed to strengthen the service, such as improved collaborations, improved drug management to increase adherence to treatment and continuous professional development to increase confidence levels in provision of NIMART, as some nurses are not NIMART trained. Nachega et al.^25^ also found that optimal treatment adherence may be difficult in pregnant and immediate postnatal women. Therefore, non-medical people such as lay or peers could play a significant role in enhancing adherence. It seemed that the revival of the buddy system as suggested by the nurses would address this challenge. Nurses believed that effective management of HIV in the postnatal period actually begins during pregnancy if women attend regular facilities and that testing should be extended to WBOTs.

## Limitations

This study did not include all nurses in this district; it was conducted at one municipality in the Free State Province. Findings of this qualitative study cannot be generalised to all PHC nurses within the province because the contexts are different. Confirmatory evaluations of the quality of care and impact of contextual factors on implementation of HIV care would have added deeper understanding. Nurses who were not NIMART trained were excluded; their understanding and implementation of guidelines may have been different.

## Conclusion

Nurses’ interpretation and implementation of public policy, in this case the national HIV guidelines, remains critical in the effective management of HIV in PHC facilities. In this study, perceptions of nurses of guidelines and their implementation practices were highlighted. The findings demonstrate that nurses had various understandings of the guidelines that provided for their practices and they implemented them with various levels of success. No gaps were found between the guidelines, their interpretations and practices. Their nursing background supported instances where patients presented with complex needs.

The management of HIV-positive mothers and HIV-exposed infants can be complex in already-overburdened health facilities. However, nurses provided the postnatal service to the best of their ability irrespective of the low resources, poorly coordinated referral systems and other challenges. Nurses articulated some successes in the reduction of vertical transmission and patient outcomes. However, in the absence of effective health data management resulting from uncoordinated referral systems, proper evaluation of PMTCT remains elusive.

Effective management of PNC requires multidisciplinary collaborations, enhancing community-based, family-centred care, increasing the clients’ role in self-management, capacity-building, adequate resources, comprehensive infant testing, effective health data management to link CD4 monitoring to HIV PCR tests and adequate maternal HIV care.
